# Implementation, evaluation, and recommendations for extension of AHRQ Common Formats to capture patient- and carepartner-generated safety data

**DOI:** 10.1093/jamiaopen/ooy004

**Published:** 2018-04-03

**Authors:** Sarah Collins, Brittany Couture, Patricia Dykes, Jeffrey Schnipper, Maureen Fagan, James Benneyan, Aziz Sheikh, David W Bates, Margarita Sordo

**Affiliations:** 1 Columbia University, Department of Biomedical Informatics, New York, NY; 2 Columbia University, School of Nursing, New York, NY; 3 Brigham and Women’s Hospital, Center for Patient Safety, Research and Practice, Boston, MA; 4 Harvard Medical School, Boston, MA; 5 Northeastern University, HealthCare Systems Engineering Institute, Boston, MA; 6 Usher Institute of Population Health Sciences and Informatics, The University of Edinburgh, Scotland, UK; 7 Harvard School of Public Health, Boston, MA

**Keywords:** patient safety, safety reporting, patient-generated health data, standards

## Abstract

**Objectives:**

The Common Formats, published by the Agency for Healthcare Research and Quality, represent a standard for safety event reporting used by Patient Safety Organizations (PSOs). We evaluated its ability to capture patient-reported safety events.

**Materials and methods:**

We formally evaluated gaps between the Common Formats and a safety concern reporting system for use by patients and their carepartners (ie friends/families) at Brigham and Women’s Hospital.

**Results:**

Overall, we found large gaps between Common Formats (versions 1.2, 2.0) and our patient/carepartner reporting system, with only 22–30% of the data elements matching.

**Discussion:**

We recommend extensions to the Common Formats, including concepts that capture greater detail about the submitter and safety categories relevant to unsafe conditions and near misses that patients and carepartners routinely observe.

**Conclusion:**

Extensions to the Common Formats could enable more complete safety data sets and greater understanding of safety from key stakeholder perspectives, especially patients, and carepartners.

## INTRODUCTION

Safety reporting systems are essential for understanding patient safety issues and supporting a “Just Culture” of learning from adverse events for continuous improvement.^1–3^ Most safety data used for safety and quality improvements within health organizations and across Patient Safety Organizations (PSOs) are clinician-reported, and underestimate true harm rates.^4^ Patient-generated health data (PGHD) increasingly are recognized as an important data source.^5^ Patient-generated health data that captures patient-reported safety concerns could be useful to increase the completeness of patient safety event data by including the patient and carepartner (eg family members or friends involved in a patient’s care) perspective of what comprises an unsafe condition, near miss or incident, and how frequently those events are experienced.^6^

Capturing safety concerns from patients in real-time is still in its infancy, with only a few known applications that have been developed and piloted.^7^^,^^8^ Standard formats are necessary to leverage information and knowledge from safety event data pooled across individuals, settings, and health organizations. Capturing safety event data from patient/carepartner perspectives in standard formats will allow cross-referencing with clinician-reported data to compare perspectives, reporting rates, and track individual events or types of events. Such cross-referencing activities will be much easier if the data elements used for reporting by different roles (eg patients, carepartners, clinicians) map to each other.

The Common Formats published by the Agency for Healthcare Research and Quality (AHRQ) represent a major step forward and are the standard used and endorsed by PSOs to enable sharing and learning from de-identified data sets of safety events that occur in the clinical setting. The Common Formats standard primarily targets clinician-reported safety events and captures 3 types of safety events: unsafe conditions, near misses, and incidents.^9^ The Common Formats defines an “incident” as a safety event that reached the patient, “a near miss” as a safety event that did not reach the patient, and an “unsafe condition” as a circumstance that increases the probability of a safety event.^9^ While the Common Formats standard does not exclude patient-reported safety data from being modeled to adhere to its format, we believe significant gaps serve as barriers to using this standard to adequately capture safety events reported by patients and their carepartners.

Problems with the validity and reliability of clinician-reported safety data are well-documented; even still, clinicians’ professional training likely provides a baseline knowledge and literacy of patient safety concepts that should inform *some* level of accuracy and reliability in safety reporting. Patients and their carepartners, as a group, do not receive comparable professional training. Therefore, content and functional specifications for a patient/carepartner reporting system will vary from content and functional specifications for clinician reporting systems. These variations require evaluation of current standards capability to adequately capture patient/carepartner-reported safety events to enable aggregation with safety data from other sources (eg clinicians).

We used the Common Formats to model the data captured in an existing electronic patient/carepartner safety reporting system used at Brigham and Women’s Hospital called MySafeCare.^10^^,^^11^ MySafeCare was developed based on other safety reporting literature and systems^3^^,^^12^^,^^13^ and used an iterative user-centered design process. MySafeCare includes features such as patient/carepartners’ ability to submit anonymously and provides 9 safety concerns categories for user selection (see Table 1). To promote use of MySafeCare in the hospital, in-person engagement rounds are conducted with patient/carepartners’ explaining “we would like to hear from you through MySafeCare to improve reporting of concerning or worrisome events.”

**Table 1.** MySafeCare data elements with match, partial match, or missing in AHRQ Common Formats v1.2 and 2.0

MySafeCare data elements	AHRQ Common Formats	
	V1.2	V2.0
Metadata		
Organization OID	Match	Match
PSO ID	Match	Match
Subject ID	Match	Match
Submission date/time	Match	Match
Clinical unit	Match	Match
Submitter and submission information		
Would you like to tell us your name and room number or stay anonymous?	Match	Missing
What is your name?	Match	Missing
What is your room number?	Missing	Missing
What is your relationship with the patient?	Match	Missing
Is your family/friend engaged in your care?	Missing	Missing
On a scale of 1–3, with 3 being the most worried, how worried are you about the unexpected or concerning event(s) that you experienced?	Missing	Missing
When did this unexpected or concerning event occur?	Match	Match
Did you share your concern with your care team?	Partial	Missing
Do you plan to share your concern?	Missing	Missing
Concern categories		
My plan	Partial	Partial
My communication	Partial	Partial
My privacy	Partial	Partial
My pain	Partial	Partial
My waiting time	Partial	Partial
My medication	Match	Match
My room	Match	Match
My hygiene	Match	Match
Other	Match	Match
Concern subcategories		
I/my carepartners do not know my plan of care	Missing	Missing
I/my carepartners feel like my care team is not following my plan of care	Missing	Missing
I/my carepartners feel there is a problem with my plan of care	Missing	Missing
I/my carepartners have other treatment concerns	Missing	Missing
I was given the wrong medication or dose^a^	Partial	Missing
I was almost given the wrong medication or dose^a^	Partial	Missing
I was not given my medication on-time	Match	Match
I missed a medication	Match	Missing
I/my carepartners have other medication concerns	Match	Missing
My medical device is not working	Missing	Missing
My medical device will not stop beeping	Missing	Missing
My medical device seems excessive	Missing	Missing
My room is not clean	Missing	Missing
I/my carepartners have other medical device concerns	Missing	Missing
I/my carepartners do not feel respected	Missing	Missing
My and/or my carepartners needs are being ignored	Missing	Missing
I/my carepartners am/are concerned about the communication between my care team about my plan of care	Missing	Missing
I/my carepartners am/are concerned about how my care team communicates with me about my plan of care	Missing	Missing
I/my carepartners have other communication concerns	Missing	Missing
A member of my care team did not wash his/her hands	Missing	Missing
A member of my care team did not wear gloves	Missing	Missing
A member of my care team did not follow the precautions on the door	Missing	Missing
I/my carepartners have other infection concerns	Missing	Missing
My and/or my carepartners privacy is/are being ignored	Missing	Missing
I/my carepartners have other privacy concerns	Missing	Missing
I/my carepartners feel that my care team is not managing my pain to my expectations	Missing	Missing
My pain is well controlled but I/my carepartners am/are concerned about the medication	Missing	Missing
Nobody has asked me or my carepartners if I am in pain	Missing	Missing
I/my carepartners have other pain management concerns	Missing	Missing
I am waiting too long for help going to the bathroom	Missing	Missing
I am waiting too long for help turning and moving in bed	Missing	Missing
I am waiting too long for my procedure	Missing	Missing
I am waiting too long to be transferred	Missing	Missing
I am waiting too long to be discharged	Missing	Missing
I/my carepartners have other waiting time concerns	Missing	Missing
Narrative content		
Please describe the event in your own words:	Match	Match
Follow-up documented by clinician	Match	Missing
Optional background information		
What is your age?	Match	Match
What is your gender?	Match	Partial
If other, please specify:	Missing	Missing
Was the admission to the hospital planned or urgent/emergent?	Missing	Missing
What is your race? (please choose one or more)	Match	Match
If other, please specify:	Missing	Match
Are you Hispanic or Latino?	Match	Match
What is your preferred language?	Missing	Missing
If other, please specify:	Missing	Missing
What is your ZIP code?	Missing	Missing
What is the highest level of education you have completed?	Missing	Missing
How often do you need to have someone help you when you read instructions, pamphlets, or other written material from your doctor or pharmacy?	Missing	Missing
Count of extra fields from AHRQ Common Formats not present in MySafeCare	189	80

a Recommended revisions to MySafeCare to align with Common Formats.

We evaluated and quantified the gaps between Common Formats and MySafeCare, and drawing on this analysis we sought to make recommendations for extensions to the standard to capture patient-/carepartner-reported safety data. This work is part of a large study investigating patient/carepartner safety reporting which has been approved by our organization’s Institutional Review Board.

## METHODS

The study team utilized 3 steps to validate mapping of concepts from MySafeCare into AHRQ Common Formats: (1) individual mapping of concepts (author: S.C.); (2) group consensus with clinical patient safety experts for mapping and interpretation of patient/carepartners reported safety data and concepts (authors: S.C., J.S., and P.D.); and (3) iterative confirmation of mapping with team member experienced in information modeling for validation of semantic consistency and modeling approach (authors: M.S., S.C.). In 2017, AHRQ updated the version 1.2 of the Common Formats (v1.2) by releasing version 2.0 (v2.0), based on feedback from the PSO community, which specified a smaller core data set that will be used for national aggregation and analysis. Analyzes were completed for both v1.2 and the v2.0.

During the mapping and analysis steps described above, we attempted to model MySafeCare data elements as comprehensively as possible without risking the integrity of the data, as judged by an information modeling expert (M.S.). We identified modifications to MySafeCare when it would improve data capture without anticipated negative impacts on patient/carepartner usability based on our user-centered design findings. To quantify the gap between MySafeCare and the Common Formats, we used codes consistent with Fifth Message Understanding Conference (MUC-5) Evaluation Metrics to categorize each MySafeCare data element as match, partial match, conflicting, extra, or missing from the Common Formats.^14^ Finally, we implemented a Clinical Document Architecture (CDA) XML and schema using the Common Formats generic and medication modules from quality reporting CDA release 2.0 using sample MySafeCare data and provide screen shots.

## RESULTS

**Figure 1.**
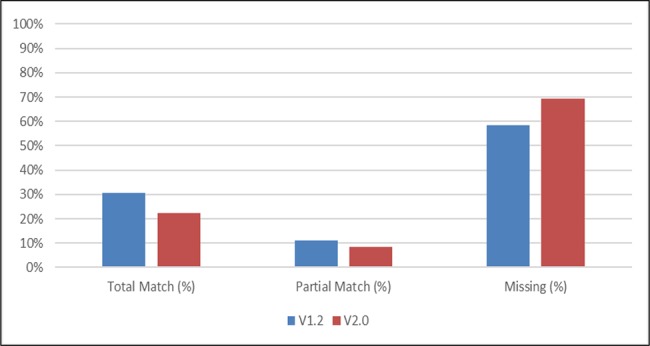
Agency for Healthcare Research and Quality Common Formats v1.2 and 2.0 Mapping to MySafeCare Patient and Care Partner Reporting System.

The revised v2.0 of the Common Formats resulted in a decrease in the number of data elements fields within MySafeCare that could be mapped using the standard from 30% in v1.2 to 22% in v2.0 (see Figure 1). Partial matches also decreased from 11% to 8% with the release of v2.0. The number of MySafeCare fields that could not be mapped to the Common Formats (ie missing) increased from 58% in v1.2 to 69% in v2.0.

Data elements to capture “submitter and submission information” dropped from 5 of 9 matching or partial match in Common Formats v1.2 to only 1 data element matching in v2.0. Matches for v2.0 and v1.2 to MySafeCare remained the same for Concern Categories, but level of match for subcategories and demographics decreased. For example, we could no longer capture that data were submitted anonymously, the submitters relationship to the patient (eg is the patient, or is a family member), and some answer choices such as “other” gender (ie not male, female, or unknown).

**Figure 2.**
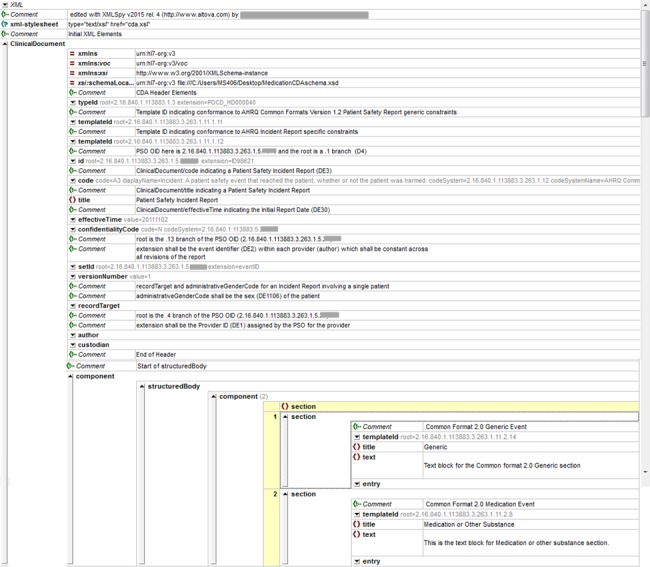
General view of the medication Clinical Document Architecture showing header and 2 sections: generic and medication.

**Figure 3.**
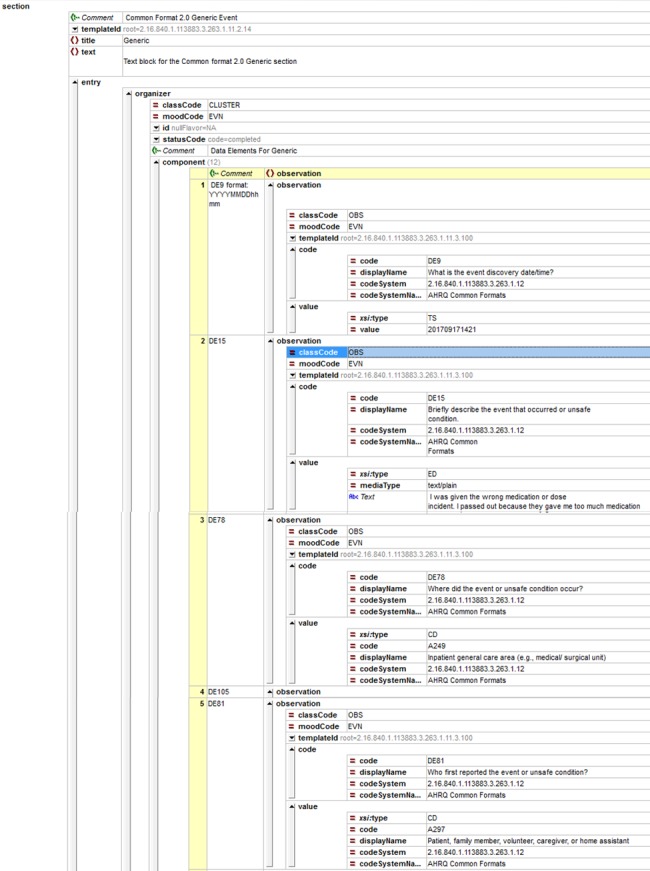
Generic section expanded of Clinical Document Architecture schema.

We identified the need to disambiguate 2 MySafeCare data elements, an incident and a near miss, in which “wrong medication” and “wrong dose” were combined in the answer choices (see Table 1). The CDA schema can be seen in Figures 2 and 3.

## DISCUSSION

Release of v2.0 of AHRQ Common Formats decreased the total number of data elements in the Common Formats significantly, and our analyzes indicated that this resulted in decreased capability to map MySafeCare data elements. Overall, we identified a “narrowing” of concepts captured from v1.2 to v2.0 that further restricted the reporting options that were available in v1.2 for non-clinical people (ie patient/carepartner) to report a safety issue. While v1.2 could not map all MySafeCare concepts, it included more options than v2.0. Overall, only 22% of concepts could be mapped with the latest version, suggesting that the standard needs to be extended.

The Common Formats include a generic module and an event specific module, with the generic section required for all types of reports. We found that all concepts in our patient/carepartner safety reporting application that could be modeled using the Common Formats were part of the generic module, except medication concerns. We could model MySafeCare medication concerns using the Common Formats medication module and note that MySafeCare could be extended to capture more detailed medication information, such as the medication name. MySafeCare was developed for ease of use by patients/carepartners and to identify *types* of safety concerns, which is why this level of medication detail was not specified, but may be desired as we seek to align data sources and increase utilization of PGHD. However, we note that interpretation of some concerns, particularly medication concerns, submitted by patients/carepartners may not be equivalent to a clinicians’ incident report. For example, clinician-reported incident of a late (or early) medication is likely only submitted when it is clinically significant and potentially harmful (eg antibiotic prophylaxis for prevention of surgical site infection). A patient-reported concern that a medication was late may not consider that the administration time was still clinically appropriate (eg daily aspirin received an hour late). Importantly, we still want to capture all patient/carepartners’ concerns, but need to ensure our reporting standards allow for both differentiating and pooling these data appropriately.

AHRQ Common Formats can have one or more event categories in a report (fall event and medicine event), but there are constraints associated with each report type (near miss, incident, or unsafe condition). For example, a fall event should not be included in a near miss or unsafe condition report, whereas a medication event may be included in an incident, near miss or unsafe condition report. Additionally, MySafeCare captured the “date/time” of the submission (which can be captured using the CDA in the generic module) but MySafeCare also captures relative dates by asking if the event occurred today, yesterday, or more than 2 days ago. The phrasing of these questions was carefully considered and tested for usability and comprehension by hospitalized patients. However, the CDA captured more “punctual” events, rather than “events in an interval” as MySafeCare did, since it covered relative dates within a full hospital stay. Therefore, we chose to use one report per event, given report type constraints and that all events are unlikely to happen on the same date. This approach provided more specific capture of metadata per safety event, but required extra processing.

Using a standard that integrates the 2 types of data (clinician-reported and patient/carepartner-reported) and facilitates interpretations based on data provenance could be beneficial for improved measuring, monitoring, and quality improvement initiatives and might lead to more robust signal detection of emerging patterns of safety issues. For example, the ability to detect if clinicians and patients/carepartners identify issues that apply to unique domains could highlight major gaps in current safety initiatives that are only based on clinician-reported data. Alternatively, the ability to identify if clinicians and patients/carepartners identify different issues within the same domain could be particularly useful to better understand complex domains, such as discharge planning and communication that likely require multi-faceted interventions for successful and impactful change.

Based on our analyzes, we propose 4 broad recommendations for extension to AHRQ Common Formats Standard with the intent to capture more comprehensive safety data inclusive of patient/carepartners’ perspectives (discussed below). These recommendations should serve as guidance for initial revisions to the Common Formats and should be refined through continued validation with other patient and carepartner safety event reporting systems.

### Recommendations for extension to AHRQ common Formats standard

1. Extend model to capture whether data reflects patient, carepartner, or clinician reporting to allow for differentiating and pooling data appropriately.
2. Capture more complete information about the submission than is captured in current versions of the standard, including if data was reported anonymously.
3. Revise value sets to include additional relevant values when answered from patient or carepartner perspective, such as “other” gender.
4. Extend categories that capture observations from patients and carepartners related to safety, particularly categories for events types of unsafe conditions and near misses.

### Limitations

This evaluation and modeling is based on the MySafeCare system, developed using user-centered design to capture patient/carepartner perspectives of safety events. Other patient safety reporting systems should be evaluated for further recommendations to extend Common Formats.

## CONCLUSION

Providing patients/carepartners with an easy, electronic mechanism to directly share and record safety concerns and events with clinicians and hospital administrators in real-time is necessary, but not sufficient, to advance our understanding of safety threats. These data must be captured in a standard format to enable identification of system weaknesses, continuous learning, and evaluation of interventions at scale. Most standards need iterative refinement, and this relatively new standard is no exception. We recommended 4 extensions to AHRQ Common Formats to enable PSOs to capture safety events reported from the patient/carepartner perspective to increase completeness and understanding of safety from multiple stakeholder perspectives. Future work should continue to validate relevant data to capture from patients/carepartners to inform patient safety improvements and enable pooling and sharing of safety data sets for continuous learning.

## FUNDING

Funding provided by the Agency for Healthcare Research and Quality (AHRQ) 1P30HS0235335 Making Acute Care More Patient Centered. The content is solely the responsibility of the authors and does not necessarily represent the official views of the Agency for Healthcare Research and Quality.

*Conflict of interest statement*. None declared.

## CONTRIBUTORS

All authors have contributed sufficiently and meaningfully to the conception, design, and conduct of the study; data acquisition, analysis, and interpretation; and/or drafting, editing, and revising the manuscript.
